# Fiber Optic Micro-Hole Salinity Sensor Based on Femtosecond Laser Processing

**DOI:** 10.3390/nano15010060

**Published:** 2025-01-02

**Authors:** Chen Li, Chao Fan, Hao Wu, Xxx Sedao, Jiang Wang

**Affiliations:** 1 College of Mechanical and Electrical Engineering, Shaanxi University of Science and Technology, Xi’an 710021, China; 2 College of Mechanical Engineering, Xi’an Jiaotong University, Xi’an 710049, China; 3 State Key Laboratory for Manufacturing Systems Engineering, Xi’an Jiaotong University, Xi’an 710054, China; 4 Laboratoire Hubert Curien, UMR CNRS 5516, Université de Lyon, 92 Rue Pasteur, CS 30122, 69361 Lyon Cedex 07, France; 5 School of Artificial Intelligence, Optics and Electronics (iOPEN), Northwestern Polytechnical University, Xi’an 710072, China

**Keywords:** femtosecond laser, Fabry–Perot, micro-hole

## Abstract

This study presents a novel reflective fiber Fabry–Perot (F–P) salinity sensor. The sensor employs a femtosecond laser to fabricate an open liquid cavity, facilitating the unobstructed ingress and egress of the liquid, thereby enabling the direct involvement of the liquid in light transmission. Variations in the refractive index of the liquid induce corresponding changes in the effective refractive index of the optical path, which subsequently influences the output spectrum. The dimensions and quality of the optical fiber are meticulously regulated through a combination of femtosecond laser cutting and chemical polishing, significantly enhancing the mechanical strength and sensitivity of the sensor’s overall structure. Experimental results indicate that the sensor achieves salinity sensitivity of 0.288 nm/% within a salinity range of 0% to 25%. Furthermore, the temperature sensitivity is measured at a minimal 0.015 nm/°C, allowing us to neglect temperature effects. The device is characterized by its compact size, straightforward structure, high mechanical robustness, ease of production, and excellent reproducibility. It demonstrates considerable potential for sensing applications in the domains of biomedicine and chemical engineering.

## 1. Introduction

Salinity is one of the most important parameters of seawater, and its accurate measurement is of great significance for marine biochemical analysis, fishery production, and marine environmental monitoring [1,2,3]. At present, salinity sensors are still mainly based on traditional electrical sensors, which determine the salinity of seawater by measuring its conductivity or resistivity [4]. However, electrical salinity sensors have the disadvantages of being large in size, susceptible to electromagnetic interference and seawater corrosion, and requiring regular calibration and maintenance [5]. Compared with traditional electrical salinity sensors, fiber optic salinity sensors have the advantages of being resistant to electromagnetic interference, corrosion-resistant, compact in structure, and highly sensitive, and they have attracted widespread attention from researchers [6]. Common types of fiber optic salinity sensors include fiber Bragg grating (FBG) [7] sensors, surface plasmon resonance (SPR) [8,9] sensors, Fabry–Perot interferometer (FP) [10] sensors, Michelson interferometer [11] sensors, and Mach–Zehnder interferometer (MZI) [12] sensors. Among them, FBG salinity sensors have low sensitivity [13] and need to be combined with sensitive materials [14], which makes them inapplicable to high-precision measurement. Although SPR salinity sensors have improved sensitivity [15], they are prone to metal oxidation [16], and the preparation process is complicated and costly. Interference salinity sensors have attracted great attention for their simple structure and high sensitivity. Fiber optic interferometry measures seawater parameters by tracking the wavelength shift of the spectral interference dip through changes in the refractive index (RI) of seawater [17].

For these different sensing configurations, a trade-off needs to be achieved between sensitivity and mechanical strength [18]. Research has indicated that an improvement in a sensor’s sensitivity will inevitably result in a decrease in its mechanical durability or speed of response. Microchannel-based interferometric sensors, which create microchannels inside optical fibers, offer a useful solution to these issues. Typically, focused ion beams [19], femtosecond laser ablation [20], and chemical etching [21] are employed to create microchannels in optical fibers. Compared with the other two processing methods, the microchannels fabricated by femtosecond laser direct ablation are conical in shape, with a simple preparation process and high preparation efficiency. It is possible to fabricate vertical microchannel structures with larger diameters in optical fibers without affecting other parts of the optical fibers, effectively retaining the mechanical strength of the optical fibers themselves. However, the inner surface of the microchannel structure prepared in the optical fiber by femtosecond laser direct ablation is relatively rough, the fiber insertion loss introduced is large, and the diameter of the microchannel in the optical fiber is uneven.

In recent years, the method of femtosecond laser-induced water breakdown has been used to prepare optical fiber microchannels [22,23]. The cavitation damage of the optical fiber material is caused by the shock waves and high-speed jets generated in the water, which effectively avoids the shortcomings of femtosecond laser processing itself. These structures have significant mechanical strength and are able to withstand lateral loads and strains while being composed of hollow chambers inside the optical fiber. Because there is no film- or polymer-integrated sensor structure, these types of sensors can also withstand high temperatures. Furthermore, long-term stability is a critical factor for practical and in situ applications of these sensors. Previous studies [18,24,25] have demonstrated the ability of femtosecond laser-fabricated microstructures in fibers to maintain structural and functional integrity over extended periods, which highlights their suitability for real-world monitoring scenarios.

This work adopts a femtosecond laser-induced water breakdown single scan method to prepare a single vertical microchannel in an optical fiber. It only needs to scan the laser focus once from the upper surface to the lower surface along the radial direction of the optical fiber to prepare a single vertical microchannel with good parameters in the optical fiber. The microchannels prepared in the optical fiber using this method have a uniform diameter, precisely controllable size and position, and low inner surface roughness. The entire preparation process only takes 1–2 min, which greatly improves the preparation efficiency. Compared with similar sensors, the sensitivity and processing efficiency are also significantly improved [26,27].

## 2. Sensor Structure Design and Working Principle

**Figure 1:**
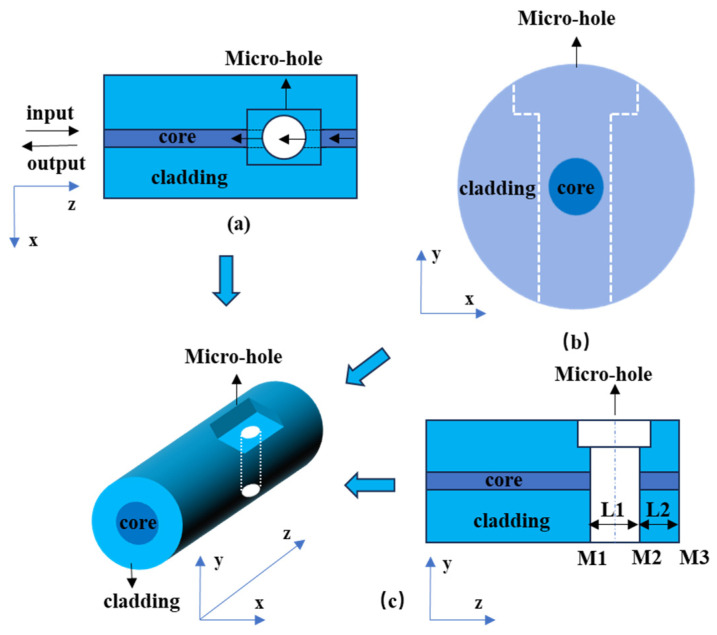
Sensor structure diagram: (**a**) top view, (**b**) side view, (**c**) front view.

Figure 1 presents a schematic of the operational principles of the sensor. The apparatus is wholly fabricated from quartz optical fiber. The micro-hole is formed by femtosecond laser processing on the side of the SMF to allow liquid to flow in and out of the fiber core. Due to the different refractive indices of solids and liquids, Fresnel reflection occurs at the liquid–solid interface, forming three reflection surfaces on the sensor head, which are denoted as M_1_, M_2_, and M_3_. Reflection surfaces M_1_ and M_2_ form an air FP cavity, denoted as Cavity1, and reflection surfaces M_2_ and M_3_ form a fiber FP cavity, denoted as Cavity2. Cavity3 consists of reflection surface M_1_ and reflection surface M_3_. The lengths of Cavity1, Cavity2, and Cavity3 are L_1_, L_2_, and L_1_ + L_2_, respectively. Considering the lower limits of the thermo-optic coefficient, which is measured at 6.3 × 10^−6^/°C, and the thermal expansion coefficient, recorded at 0.55 × 10^−6^/°C, for the optical fiber, the refractive index n_2_ and the lengths of the two FP cavities, L_1_ and L_2_, can be considered constants within the typical operating temperature range of the sensor.

From Figure 1, it is apparent that the micro-hole on the fiber core serves as both a scatterer and a non-standard FP cavity. In Figure 1c, the stepped micro-hole represents a model of an FP cavity. As light propagates through this optical fiber, it is inevitably affected by the modulation of the FP cavity. A comparison of Figure 1a,c reveals distinct differences in how the FP cavity resonance affects the system. Specifically, while the resonance primarily influences the y-z plane in Figure 1c, Figure 1a captures a broader interaction, including both the scattering effect and the FP cavity resonance across multiple planes. This broader interaction in Figure 1a provides a more comprehensive representation of the system’s behavior. Therefore, Figure 1a is more suitable for analyzing the primary characteristics of the original three-dimensional problem. Based on this observation, the subsequent two-dimensional analysis model in this study will be developed.

## 3. Simulation

### 3.1. Reflection Spectrum Characteristics at Different Apertures

**Figure 2:**
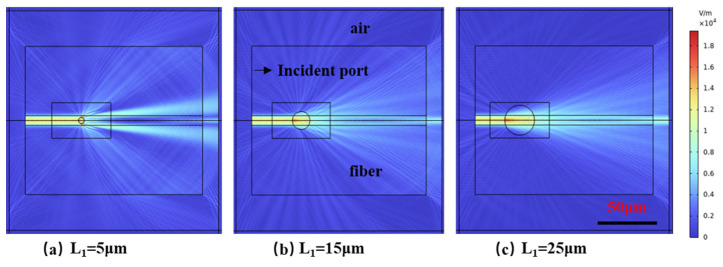
Electric field distribution under different apertures: (**a**) L_1_ = 5 μm, (**b**) L_1_ = 15 μm, (**c**) L_1_ = 25 μm.

We use the COMSOL6.2 simulation software to analyze the problem in Figure 1a, mainly analyzing the TE wave [28] in detail. In this instance, the orientation of the Ey component is aligned with the axial direction of the micro-hole and is perpendicular to the working surface. Using 1550 nm light wave excitation, the distribution of the Ey component of the guided wave passing through micro-holes with diameters of 5 µm, 15 µm, and 25 µm is simulated, and the simulation results are shown in Figure 2. The model domain consists of the optical fiber and the surrounding air, with the absorption boundary defined by the widely used anisotropic perfect matching layer (APML). The APML is a numerical technique employed to simulate an open boundary in wave propagation problems. Unlike isotropic layers, the anisotropic nature of the APML allows it to more effectively absorb incident waves in different directions, minimizing reflections at the boundaries. This makes the APML particularly suitable for problems involving complex geometries and varying material properties, such as the present study. Since this waveguide supports only the lowest-order mode, its mode field distribution can be readily calculated using the effective refractive index method. The effective refractive index method is a widely used approach for the approximation of the propagation characteristics of waveguides, particularly when only the fundamental mode is considered. In this method, the waveguide is treated as an effective homogeneous medium with an ‘effective’ refractive index, which is derived from the physical properties of the waveguide, such as its geometry and the refractive indices of the core and cladding materials. This simplification allows for the efficient calculation of the mode field distribution without solving the full waveguide mode equation. In order to examine the scattering effects produced by the micro-hole on the guided wave, as well as the progression of the transverse electric field distribution along the direction of propagation, the optical fiber is configured to a length of 200 µm. The length L_2_ of Cavity2 is specified as 100 µm, and the refractive index of the material filling the micro-hole is established at 1.

As can be seen from Figure 2, the scattering and recoupling of the scattered wave are closely related to the micro-hole diameter. The values of the scattering loss and transmittance changing with the micro-hole diameter are shown in Table 1.

**Figure 3:**
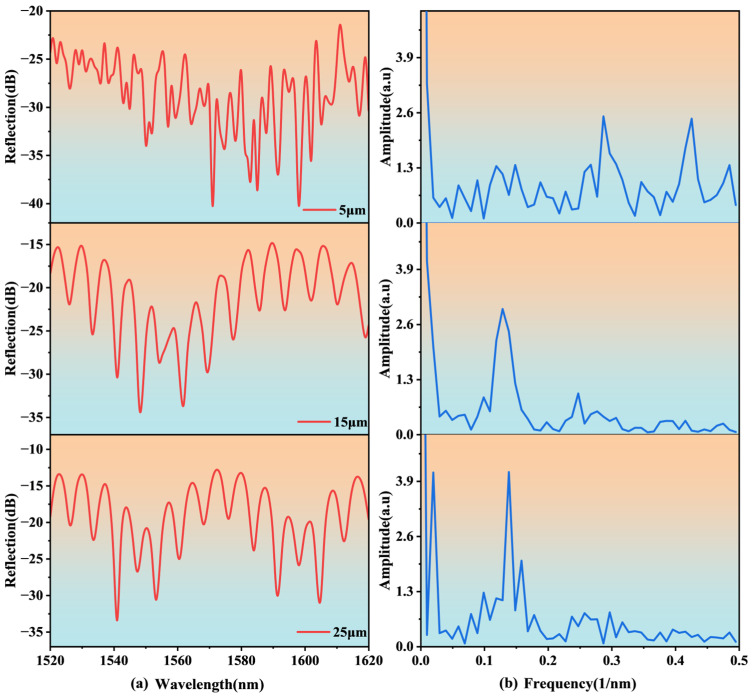
Spectral characteristics of the sensor: (**a**) reflection spectra, (**b**) frequency spectra.

As can be seen from Table 1, as the micro-hole diameter increases, the transmittance in the fiber core increases and the scattering loss decreases. This is mainly attributed to the modulation of the light field distribution and mode coupling by the aperture. When the aperture is small, the main energy of light is concentrated at the edge area of the micro-hole, and the edge surface roughness and refractive index gradient will cause strong scattering and reflection, resulting in higher energy loss. As the diameter of the through hole increases, the mode field expands, and the energy distribution of the light gradually moves away from the edge area, significantly weakening the scattering and reflection effects of the boundary. In addition, after the geometric constraints at the edge are weakened, the light field is distributed more uniformly within the micro-hole and the propagation path is more stable, thereby reducing additional losses caused by multiple scattering and interference coupling. Finally, the reflection spectrum is analyzed, and a light wave with a wavelength range of 1520 nm~1620 nm is used for excitation. The reflection spectrum distribution can be obtained by integrating the Poynting vector of the light wave reflected back to the boundary of the incident port. The reflection spectrum of the cascaded FP structure is formed by the superposition of the spectra of each cavity. In order to better analyze the characteristics of the interference fringes, it is also necessary to perform Fourier transform or spatial frequency domain analysis on the measured wavelength domain spectrum. The simulation results are shown in Figure 3.

As shown in Figure 3, when the aperture is 5 µm, both the reflection spectrum and the frequency spectrum appear chaotic, with no distinct interference fringes present, highlighting the more pronounced scattering effect. When the aperture increases to 15 μm, the reflection spectrum begins to show clearer interference fringes, mainly including a fast-changing interference fringe from Cavity2 (named as the sensing cavity) and a slowly changing interference fringe from Cavity1. Since the length of Cavity2 is much longer than that of Cavity1, the interference fringes generated by Cavity1 only serve as the envelopes of the interference fringes generated by Cavity2. However, it can be observed from its spectrum that there is only one characteristic peak, so the interference fringes are mainly modulated by Cavity2 at this time. This is also expected in subsequent experiments. When the aperture is further increased to 25 μm, the resonance effect is more obvious, and two characteristic peaks appear in its spectrum; the first peak corresponds to Cavity1, and the second peak corresponds to Cavity2. This is because the transmittance and aperture of the micropores in the fiber core increase; more light is recoupled into Cavity2, and Cavity1 and resonates. Therefore, the interference fringes are mainly affected by the combined effects of Cavity1 and Cavity2.

Therefore, considering the mechanical strength of the sensor and its reflection spectrum characteristics, an aperture of 15 µm is selected as the optimal parameter for processing.

### 3.2. Reflection Spectrum Characteristics Under Different Salinities

**Figure 4:**
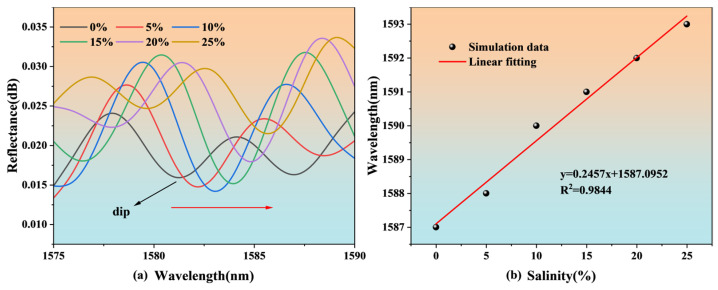
Salinity simulation results of the FP structure: (**a**) spectra shift, (**b**) linear fitting.

The simulation analysis shows the reflection spectrum characteristics of the sensor when the salinity is 0%~25% (corresponding to the refractive index (RI) of 1.3331~1.3792). The drift and linear fitting of the FP resonance dip are shown in Figure 4.

As can be seen from Figure 4a, within the salinity range of 0% to 25%, as the salinity increases, the sensor’s reflection spectrum shifts toward the long-wave direction. The trough near the wavelength of 1587 nm is selected as the observation point dip. A linear fit is performed on the change in dip, and the result is shown in Figure 4b. The linearity is 0.9844, which indicates a good linear relationship, and the sensitivity is 0.246 nm/%.

## 4. Experiment

### 4.1. Processing

**Figure 5:**
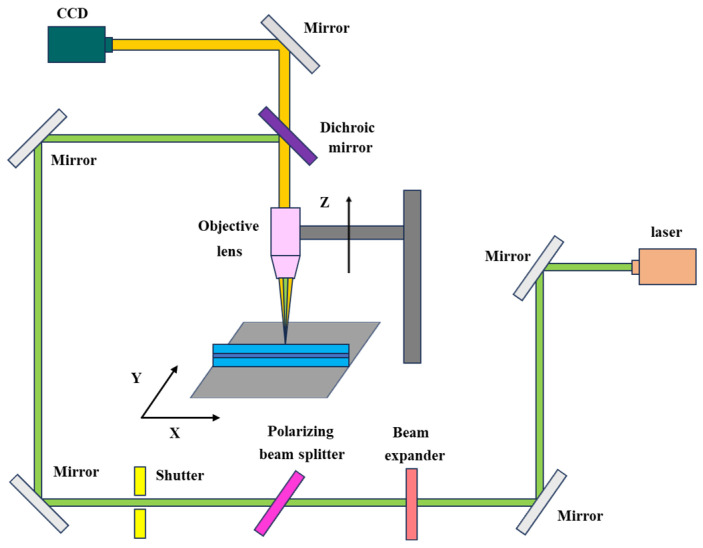
Schematic of the fs laser machining system.

In this work, micro-hole processing was performed on an ordinary single-mode optical fiber (SMF-28Ultra, Corning Incorporated, New York, NY, USA), and a refractive index matching oil (VH-521, Shanghai Xianhu Communication Equipment Co., Ltd., Shanghai, China) with a refractive index of 1.47 was used as an auxiliary liquid. The fiber laser processing system used in this study is shown in Figure 5. The femtosecond laser system is a commercial mode-locked fiber laser (FemtoYL-40, Wuhan Yangtze Soton Laser Co., Ltd., Wuhan, China), which produces 300 fs pulses (FWHM) at a wavelength of 515 nm, and the pulse repetition rate can be adjusted from 25 kHz to 5 MHz. The laser passes through a beam expander, a polarization beam splitter, and an aperture before entering a 10× objective lens (M Plan Apo SL, Mitutoyo Corporation, Kawasaki, Kanagawa, Japan). The beam expander increases the size of the laser beam, ensuring that it is properly aligned with the optical components. The polarization beam splitter separates the incident light into two orthogonal polarization components, allowing the selective control of the polarization state of the beam. The aperture is used to limit the size of the beam, ensuring that only the desired portion of the beam enters the objective lens, thereby improving the focus and precision of the optical measurements. The fiber was fixed by a fixture, and the processing trajectory and processing speed were controlled by programming the 3D micro-positioning platform. A CCD camera was used to align the sample and obtain a real-time view of the laser processing.

**Figure 6:**
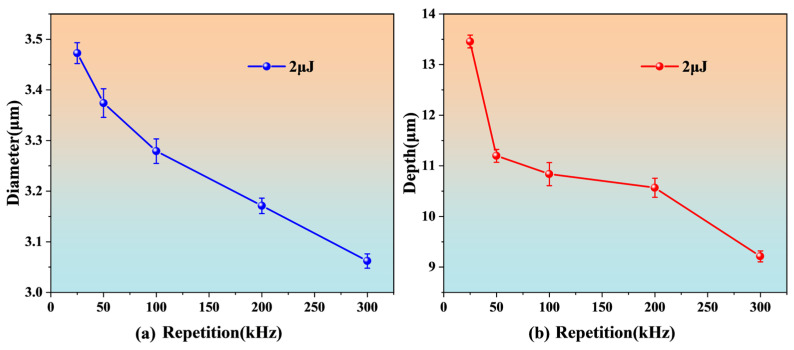
The effect of the repetition rate on micro-hole processing on the surfaces of optical fibers: (**a**) diameter, (**b**) depth.

Before processing the device, this work first discusses the effect of different repetition frequencies on the micro-hole processing on the optical fiber surface. The spot was focused on the optical fiber surface, and the changes in the optical fiber ablation aperture and hole depth were analyzed when the repetition frequency was 25, 50, 100, 200, and 300 kHz under a single pulse energy of 2 μJ and an exposure time of 1 s. The processing results are shown in Figure 6.

As can be seen from Figure 6, the diameter and depth of the fiber ablation hole decrease with the increase in the repetition frequency. This may be because, at high repetition rates, the spatter produced by material melting or vaporization may be redeposited around the hole mouth, hindering the further ablation of subsequent laser pulses. In order to obtain the maximum processing efficiency, this work selected a repetition frequency of 25 kHz as the optimal parameter for the processing of the device.

It is considered that, when processing micro-holes, the nonlinear absorption and refraction of laser by the optical fiber will further hinder the feeding of the laser in the depth direction. Therefore, before micro-hole processing, a groove is ablated on the surface of the optical fiber with a laser to minimize the loss of the laser inside the optical fiber.

**Figure 7:**
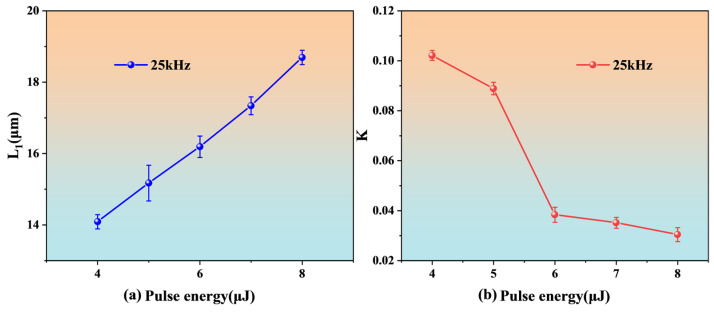
The effect of single pulse energy on Cavity1 machining: (**a**) L_1_, (**b**) K.

This work further discusses the effect of different single pulse energies on the processing quality of Cavity1, focusing the light spot at the bottom center of the groove and controlling the movement of the three-dimensional platform by computer to move the laser focus from the entrance to the exit of the cylindrical micro-hole. After about 10 s of continuous ablation, the optical fiber is pierced. The processing results are shown in Figure 7.

From Figure 7a, we can see that the length L_1_ of Cavity1 increases with the increase in the single pulse energy. When the energy is 4, 5, and 6 μJ, the processing result is close to 15 μm. However, from Figure 7b, it can be seen that only when the single pulse energy is 6 μJ, the micro-hole taper (K) near L_1_ = 15 μm reaches the minimum [K = (D − d)/H], where D is the diameter at the entrance, d is the diameter at the exit, and H is the micro-hole depth. Therefore, the repetition frequency of 25 kHz and the single pulse energy of 6 μJ are selected as the optimal parameters for the micro-hole processing of the optical fiber.

**Figure 8:**
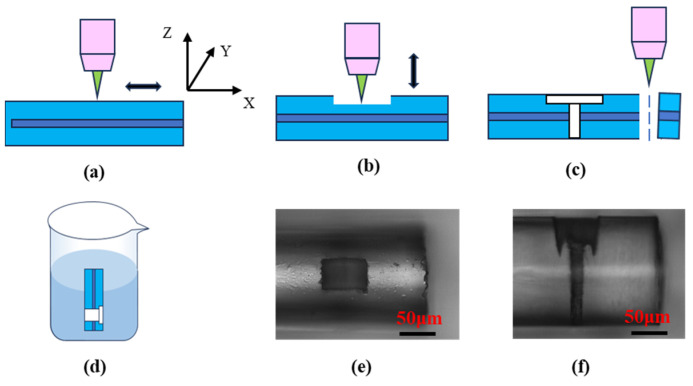
Fabrication process and optical pictures of the sensor: (**a**) Ablation groove, (**b**) Processing micro-holes, (**c**) Cutting Fiber, (**d**) Etching Fiber, (**e**) Top view, (**f**) Side view.

The specific processing steps of the sensor and its optical photos are shown in Figure 8: (a) ablating grooves on the surface of the optical fiber with a femtosecond laser; (b) processing the micro-hole in the optical fiber with a femtosecond laser; (c) cutting the optical fiber with a femtosecond laser at about 100 μm from the micro-hole; (d) immersing the processed optical fiber in a 5% HF solution for about 5 min, taking it out, and cleaning it with deionized (DI) water; (e) top view of the sensor; (f) side view of the sensor.

After processing, the diameter of the micro-hole is about 15 μm. As can be seen from Figure 8e,f, the difference between the inlet and outlet diameters is small. Since the two walls of the micro-hole are almost parallel, the difference between the inlet and outlet diameters of the micro-hole is much smaller than the depth of the micro-hole; the taper is extremely small, and the processing quality is high.

### 4.2. Test

#### 4.2.1. Salinity Experiment

**Figure 9:**
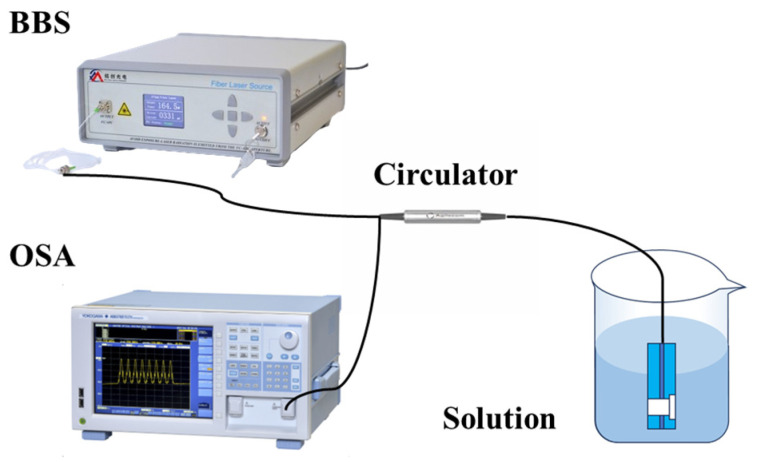
Schematic diagram of salinity device.

The reflecting seawater salinity measurement device is depicted in Figure 9. A broadband light test source (BBS) operating in the wavelength range of 1520 nm to 1630 nm is used to inject light into the sensor, and an optical spectrum analyzer (OSA) is used to gather the output spectrum. A fiber optic circulator can be used to measure the reflected output of the sensor that is being tested.

The output of every sensor is measured when it is submerged in solutions with varying salt levels in order to experimentally quantify the sensitivity of each sensor to salinity. In order to ensure the accuracy of the experimental measurements, we use a fairly large volume of solution to ensure that the experimental results are not affected by the evaporation of the solution during the measurement process. First, high-purity sea salt of different masses is weighed using a precision electronic balance, and then the corresponding volume of deionized water is slowly added and stirred until fully dissolved. Finally, the concentration is calibrated with the help of an optical refractometer. This set of salt solutions under examination has a mass percentage concentration ranging from 0% to 25%, or an RI interval of 1.3331 to 1.3792. The sensor is cleaned with DI water in between readings to eliminate the possibility of cross-contamination between solutions with varying salinities. Furthermore, to ascertain the wavelength drift brought about by the studied salt solutions, DI water is utilized as a baseline. The sensor’s repeatability is validated by its consistent return to the baseline after each rinsing cycle. The recuperation period, defined as the time required for the sensor signal to stabilize at the baseline, spans approximately three consecutive optical spectrum analyzer scans, which corresponds to about nine seconds.

**Figure 10:**
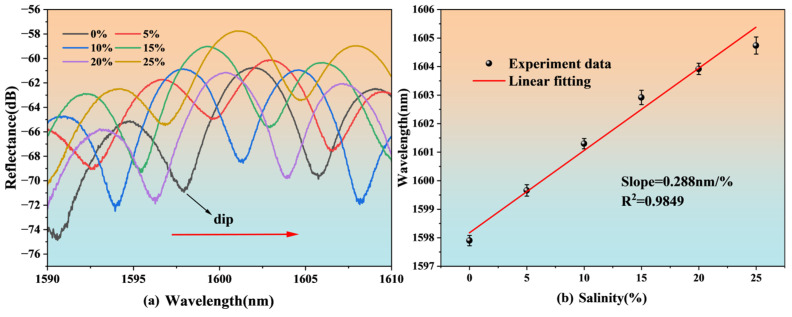
Salinity experiment results for the FP structure: (**a**) spectral shift, (**b**) linear fitting.

As shown in Figure 10a, within the salinity range of 0% to 25%, as the salinity of the seawater increases, the sensor’s reflection spectrum shifts toward the long-wave direction. The dip near the wavelength of 1597 nm is selected as the observation point dip. The change in dip is linearly fitted, and the result is shown in Figure 10b. The linearity is 0.9849, which indicates a good linear relationship, and the sensitivity is 0.288 nm/%, which is close to the simulation result. Based on the sensitivity of 0.288 nm/% and the experimental system noise level of approximately 0.05 nm, the estimated salinity resolution is 0.17%.

#### 4.2.2. Temperature Experiment

**Figure 11:**
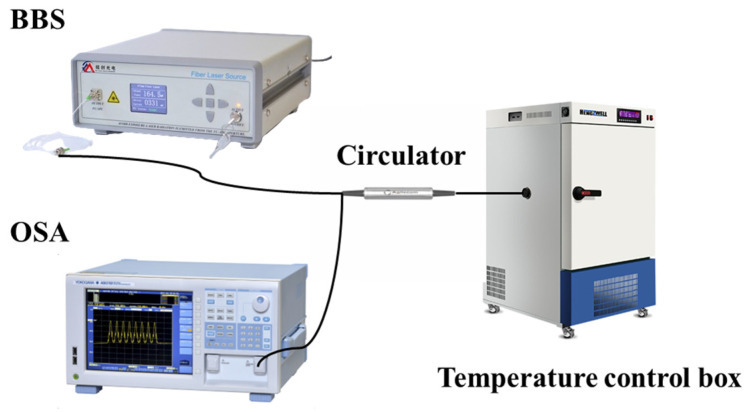
Schematic diagram of temperature device.

The temperature experiment device is connected according to Figure 11. The light is emitted by the broadband light source and transmitted to the circulator to reach the sensor. After reflection, it enters the spectrometer. The experiment uses a programmable constant temperature and humidity test box with temperature control accuracy of 0.01 °C. The temperature change range in the experiment is −5~45 °C. The spectrum change is recorded every 10 °C. When the temperature reaches the set temperature, it is kept for 15 min before recording the spectrum. We place the sensor head into the test box and tighten the furnace plug.

**Figure 12:**
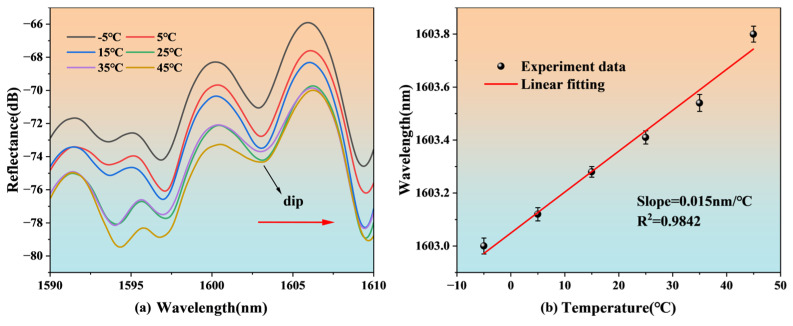
Temperature experiment results for the FP structure: (**a**) spectral shift, (**b**) linear fitting.

The spectra corresponding to the sensor changes at different temperatures are shown in Figure 12. As the temperature increases, the interference spectrum gradually redshifts, and the dip near the wavelength of 1603 nm is selected as the observation point dip. The change in dip is linearly fitted, and the result is shown in Figure 12b. The linearity is 0.9842, which indicates a good linear relationship. The sensitivity is 0.015 nm/°C. The temperature sensitivity of this sensor is low [19,29], which means that temperature crosstalk can be ignored in different types of salinity sensing. Similarly, based on the temperature sensitivity of 0.015 nm/°C and the same noise level of approximately 0.05 nm, the estimated temperature resolution is 3.33 °C.

## 5. Conclusions

In conclusion, a hollow cavity fabricated through femtosecond laser machining and wet etching has been demonstrated for salinity sensing, exhibiting good sensitivity and response speeds compared to other fiber-based sensors. This configuration is also less influenced by the temperature, featuring temperature sensitivity of 0.015 nm/°C and negligible temperature cross-sensitivity, which are critical for in situ sensing applications. The innovative fabrication process is straightforward and cost-effective, comprising two simple steps: femtosecond laser machining and wet etching. The sensor is characterized within a salinity range of 0% to 25%, showing sensitivity of 0.288 nm/%, effectively covering the salinity ranges of lakes, seawater, and oceans found in nature. In addition, while the current study focuses on a salinity range of 0–25% due to its relevance to most natural and marine applications, further investigation into hyper-saline or other extreme environments could provide valuable insights into the sensor’s broader applicability. The robustness of the Fabry–Perot cavity design suggests its potential for reliable performance at salinity levels exceeding 25%, warranting future experimental validation to explore these conditions. The sensor boasts advantages such as robustness, a fast response, high sensitivity, a low cost, and ease of fabrication and packaging, making it suitable for widespread use in marine monitoring.

## Figures and Tables

**Table 1:** Scattering loss and transmittance at different apertures.

Diameter	Scatter Loss	Transmittance
5 μm	0.9124	0.0874
15 μm	0.7033	0.2938
25 μm	0.5626	0.4286

## Data Availability

The data that support the findings of this study are available from the corresponding author upon reasonable request.
